# Soluble perlecan domain i enhances vascular endothelial growth factor-165 activity and receptor phosphorylation in human bone marrow endothelial cells

**DOI:** 10.1186/1471-2091-11-43

**Published:** 2010-11-03

**Authors:** Arivalagan Muthusamy, Carlton R Cooper, Ronald R Gomes

**Affiliations:** 1 Department of Orthopaedics and Rehabilitation, Penn State College of Medicine, Hershey, Pennsylvania 17033, USA; 2 Department of Biological Sciences, Center for Translational Cancer Research, University of Delaware, Newark, Delaware 19716, USA

## Abstract

**Background:**

Immobilized recombinant perlecan domain I (PlnDI) binds and modulates the activity of heparin-binding growth factors, *in vitro*. However, activities for PlnDI, in solution, have not been reported. In this study, we assessed the ability of soluble forms to modulate vascular endothelial growth factor-165 (VEGF_165_) enhanced capillary tube-like formation, and VEGF receptor-2 phosphorylation of human bone marrow endothelial cells, *in vitro*.

**Results:**

In solution, PlnDI binds VEGF_165 _in a heparan sulfate and pH dependent manner. Capillary tube-like formation is enhanced by exogenous PlnDI; however, PlnDI/VEGF_165 _mixtures combine to enhance formation beyond that stimulated by either PlnDI or VEGF_165 _alone. PlnDI also stimulates VEGF receptor-2 phosphorylation, and mixtures of PlnDI/VEGF_165 _reduce the time required for peak VEGF receptor-2 phosphorylation (Tyr-951), and increase Akt phosphorylation. PlnDI binds both immobilized neuropilin-1 and VEGF receptor-2, but has a greater affinity for neuropilin-1. PlnDI binding to neuropilin-1, but not to VEGF receptor-2 is dependent upon the heparan sulfate chains adorning PlnDI. Interestingly, the presence of VEGF_165 _but not VEGF_121 _significantly enhances PlnDI binding to Neuropilin-1 and VEGF receptor-2.

**Conclusions:**

Our observations suggest soluble forms of PlnDI are biologically active. Moreover, PlnDI heparan sulfate chains alone or together with VEGF_165 _can enhance VEGFR-2 signaling and angiogenic events, *in vitro*. We propose PlnDI liberated during basement membrane or extracellular matrix turnover may have similar activities, *in vivo*.

## Background

Perlecan, a heparan sulfate proteoglycan with preferred localization to vascular basement membranes, is comprised of a ~480 kDa protein core with five distinct domains (I - V). Domains II-V share structural homologies with other protein modules [1]. In contrast, N-terminal domain I (PlnDI) is structurally unique. As a ~22 kDa protein core, PlnDI contains 172 amino acid residues that give rise to a sperm protein, enterokinase and agrin (SEA) module localized downstream of three Ser-Asp-Gly motifs that serve as glycosaminoglycan (GAG) attachment sites [2,3].

Through the chondroitin and heparan sulfate GAG chains attached to domain I, perlecan functions as a ligand reservoir for storage, release, and protection of heparin-binding growth factors (reviewed by Whitelock et al., 2008). These interactions allow perlecan to modulate a range of biological functions, including angiogenesis (reviewed by Bix and Iozzo, 2008)[4]. Recent studies suggest immobilized forms of perlecan and PlnDI bind VEGF_165 _to coordinate developmental angiogenesis by modulating VEGF_165_/VEGFR-2 signaling [5,6]. However, a role for soluble forms of PlnDI and the mechanism(s) by which it modulates VEGF_165_/VEGFR-2 signaling is unclear.

Angiogenic activities of VEGFs are mediated primarily through two receptors [7], VEGFR-1 or fms-like tyrosine kinase 1 [8] and VEGFR-2, also known as kinase domain receptor, and fetal liver kinase 1 [9,10]. Although VEGFR-1 exhibits higher binding affinity for VEGFs, VEGFR-2 dominates VEGF induced mitogenic and angiogenic responses on endothelial cells [11,12]. VEGFR-2 signaling is enhanced by interactions with co-receptors such as heparin/heparan sulfate and Neuropilin 1 (NRP-1) [13]. In addition, VEGF binding to VEGFR-2 and NRP-1 is enhanced by exogenous heparin [14,15]. Although the natural cell surface and basement membrane polysaccharide, *in vivo*, is heparan sulfate, not heparin, few cell surface or extracellular HSPGs have been shown to modulate VEGF/VEGFR interactions [6,16].

Herein, we tested the hypothesis that soluble forms of recombinant PlnDI bind and increase VEGF_165_/VEGFR-2 interactions on human bone marrow endothelial cells, *in vitro*. Observations from this investigation suggests soluble forms of recombinant PlnDI are biologically active and capable of interacting with components of the VEGFR-2 signaling complex, enhance activity and downstream signaling related to endothelial cell angiogenic processes.

## Results

### Purification and biochemical characterization of PlnDI

Recombinant PlnDI was purified from conditioned media of HEK 293 EBNA clones as reported previously [17], and further enriched by passage through a Sepharose CL-6B column. This additional step removed high molecular weight contaminants secreted into the serum free media (i.e., full length perlecan). Aliquots of the eluted product were subsequently analyzed by SDS-PAGE and Western blotting to identify the GAG chain composition and preparation purity.

**Figure 1:**
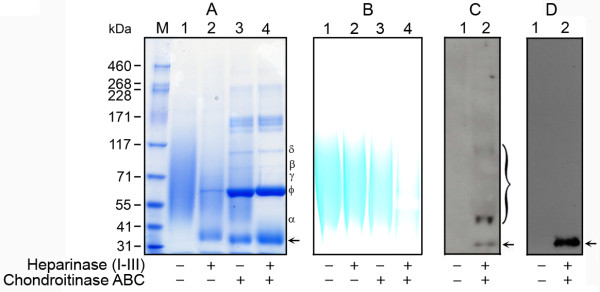
**SDS-PAGE and Western blot analysis of PlnDI**. (A) Coomassie blue staining; (B) Alcian blue staining: *lane 1*, undigested PlnDI; *lane 2*, heparinase cocktail treated PlnDI; *lane 3*, chondroitinase ABC treated PlnDI; *lane 4 *heparinase cocktail and chondroitinase ABC treated PlnDI. (C) Western blot analysis with PlnDI and (D) heparan sulfate stub (3G10) specific antibodies respectively: *Lane 1*, undigested PlnDI; *lane 2*, heparinase cocktail and chondroitinase ABC treated PlnDI. Heparinase cocktail is a mixture of heparinases I, II, and III. *Arrows *indicate the protein core of PlnDI released after heparinase cocktail and chondroitinase ABC digestion. α,β,γ,δ, ϕ indicate the migration positions of heparinases I, II, III, chondroitinase ABC, and BSA respectively. Bracket in panel C denotes immunoreactive products released following incomplete digestion.

In Coomassie blue stained SDS-PAGE gels, undigested samples displayed a broad band between ~45-117 kDa (Figure 1A, lane 1); whereas aliquots pre-treated with a heparinase cocktail yielded a distinct band at ~36 kDa_, _with a broad band between 55 -71 kDa (Figure 1A, lane 2). Chondroitinase ABC pre-digestion yielded a distinct band at ~33 kDa and broad band between 45 -117 kDa (Figure 1A, lane 3). Pre-digestion with both GAG lyases yielded a single band at 33 kDa (Figure 1A, arrow lane 4). The additional bands appearing in Figure 1A, lanes 2-4, represent BSA (ϕ, ~66 kDa), chondroitinase ABC (δ, ~100 kDa), and heparinases I (α, ~43 kDa), II (β, ~84 kDa), and III (γ, ~70 kDa).

In Alcian blue stained SDS-PAGE gels, undigested samples displayed a broad band between ~45-117 kDa (Figure 1B, lane). Aliquots pre-treated with a heparinase cocktail yielded a broad band between ~50-100 kDa (Figure 1B, lane 2). Chondroitinase ABC pre-digestion yielded a broad band between ~50-84 kDa (Figure 1B, lane 3). Pre-digestion with both GAG lyases abolished the majority staining.

The presence of PlnDI was confirmed by Western blotting using anti-PlnDI specific antibodies (CSI-0071) and antibodies (3G10) to anti-Δ-heparan sulfate that recognize heparan sulfate neo-epitopes, generated following heparinase cleavage (arrow Figure 1C and 1D). Neither antibody recognized undigested products; however, anti-PlnDI antibodies recognized partially digested products (bracket in Figure 1C, lane 2) and both antibodies recognize a distinct band at 33 kDa (arrow, Figure 1C and 1D). The 33 kDa band reflects the domain I core protein adorned with GAG chain linkage residues following heparinase digestion.

Biochemical analysis of PlnDI suggests a protein and uronic acid content of 49% and 37%, respectively (Table 1). Hexosamine (monosaccharide) compositional analysis revealed PlnDI GAGs are composed predominantly of galactosamine (60%) relative to glucosamine (40%) (Table 1). The disaccharide composition of purified PlnDI revealed 6-sulfated disaccharide as the major Δdi-CS with lesser amounts of nonsulfated and 4-sulfated disaccharides (Table 2). The major Δdi-HS derived from PlnDI was nonsulfated and Δdi-S_1 _with considerable, but lesser amounts of Δdi-S_2_, -6-sulfated, -N-sulfated, and -triS disaccharides (Table 2). The HS GAG chains on PlnDI contain approximately 3 fold more 6*-O- *than 2*-O*-sulfation.

**Table 1:** Biochemical composition of PlnDI.

	Composition (% dry weight)		Hexosamine (% mol)	
Sample	Protein	Uronic acid	GalN	GlcN
PlnDI	49 (0.37)	37 (0.29)	60 (0.87)	40 (0.88)

Data are presented as the mean of three independent experiments ± (SEM).

**Table 2:** Disaccharide composition of Chondroitin and Heparan sulfate chains of PlnDI.

	Chondroitin sulfate (% mol)			Heparan sulfate (% mol)					
Sample	Δdi-0S	Δdi-4S	Δdi-6S	Δdi-0S	Δdi-NS	Δdi-6S	Δdi-S_1_	Δdi-S_2_	Δdi-triS
PlnDI	34 (0.88)	28 (0.88)	38 (1.45)	31 (1.15)	2 (0.88)	9 (1.76)	40 (1.73)	15 (1.45)	3 (1.09)

Data are presented as the mean of three independent experiments ± (SEM).

### VEGF_165 _binds to PlnDI in a heparan sulfate dependent manner

**Figure 2:**
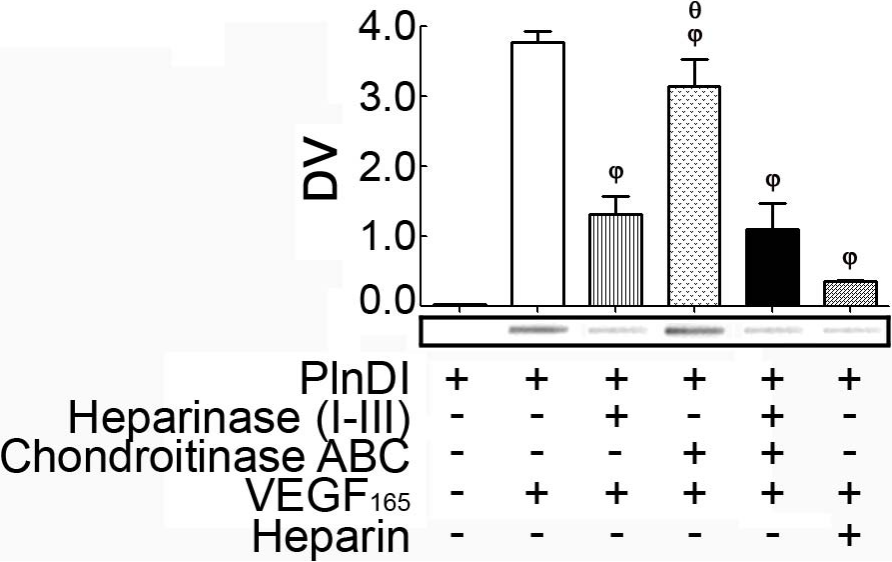
**Immunoblot analysis of VEGF_165 _binding to immobilized PlnDI**. PlnDI, undigested or pre-digested with a heparinase cocktail +/- chondroitinase ABC were immobilized on nitrocellulose and incubated with VEGF_165_. Bound VEGF_165 _was detected with VEGF specific monoclonal antibodies. Horizontal bars represent the densitometric scanning of slots outlined in rectangular boxes. Data are presented as mean density values (DV) of triplicate determinations ± SEM. (φ) different from PlnDI+VEGF_165_; (θ), different from PlnDI+Hepase+VEGF_165_. *p *< 0.05.

To identify requirement(s) for VEGF_165 _binding to PlnDI, both solid and solution phase binding assays were performed. In solid phase binding assays, immobilized PlnDI binds VEGF_165 _in a heparan sulfate dependent manner (Figure 2). Heparinase cocktail treatment of PlnDI, prior to immobilization on nitrocellulose, reduced VEGF_165 _binding by ~75% (Figure 2). In contrast, pre-digestion with chondroitinase ABC did not alter VEGF_165 _binding. Studies with the PlnDI protein core, prepared following digestion with a mixture of both enzymes, suggest VEGF_165 _poorly binds this region. VEGF antibodies do not bind immobilized PlnDI (Figure 2). In competitive inhibition assays, heparin [0.25 μg/ml] prevented ~80% of VEGF_165 _binding to PlnDI (Figure 2).

**Figure 3:**
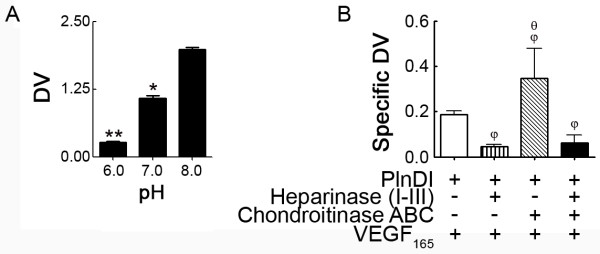
**VEGF_165 _binding to PlnDI in solution is pH dependent**. PlnDI pre-incubated with VEGF_165 _in 25 mM HEPES (pH 6.0, 7.0, or 8.0) was immobilized on nitrocellulose. VEGF_165 _bound to PlnDI was detected with VEGF specific monoclonal antibodies. (A) pH dependent binding of VEGF_165 _to PlnDI. (B) The requirement of CS and HS chains for VEGF_165 _binding to PlnDI (pH 7.0). Data are presented as mean specific density values (DV) of triplicate determinations ± SEM. Specific binding was determined by subtracting the background binding of VEGF_165 _to nitrocellulose from total bound. (*) different from pH 8.0; (**) different from pH 7.0; (φ) different from PlnDI+VEGF_165_; (θ), different from PlnDI+Hepase+VEGF_165_. *p *< 0.05.

In solution, requirements for VEGF_165 _binding to PlnDI were similar, but the capacity of binding demonstrated pH dependence (Figure 3A). When the pH of solution was reduced from 8.0 to 7.0 then 6.0, VEGF_165 _binding was reduced by 50% and 80%, respectively (Figure 3A). To identify VEGF_165 _specific binding, the background binding of VEGF_165 _to nitrocellulose was subtracted from total bound to PlnDI [18]. Employing this approach, PlnDI-HS chains account for nearly all VEGF_165 _binding, and the presence of CS chains masks VEGF_165 _interaction with HS (Figure 3B). In panel B, neutral pH was chosen to more closely reflect tissue culture conditions of subsequent experiments.

### PlnDI modulation of VEGF_165 _bio-activity

**Figure 4:**
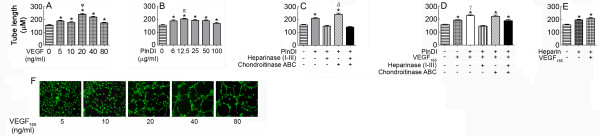
**Exogenous PlnDI enhances capillary tube-like formation**. Human bone marrow endothelial cells were seeded in 96 well plates pre-coated with growth factor reduced Matrigel and treated with PlnDI pre-incubated +/- VEGF_165_. Tube length quantification was performed after 18 h on fixed cells, stained with SYTO13. Effect of VEGF_165 _(A), and PlnDI (B) dose on capillary tube-like formation. (C) PlnDI [12.5 μg/ml] enhanced tube-like formation: Requirement of CS and HS chains. (D) PlnDI/VEGF_165 _enhanced tube-like formation: Requirement of CS and HS chains on PlnDI. (E) Effect of heparin/VEGF_165 _mixtures on capillary tube-like formation. (F) Representative images of tube-like formation in panel A. Data are presented as mean lengths of nine areas from triplicate determinations ± SEM. (*) different from media; (ψ) different from VEGF_165 _at 5, 10 and 80 ng; (π) different from PlnDI 6 and 25 μg; (δ) different from PlnDI; (γ) different from VEGF_165_. *p *< 0.05.

To identify a role for PlnDI in modulating VEGF_165 _activity *in vitro*, human bone marrow endothelial cells were employed in two independent assays: 1) VEGF_165_-enhanced capillary tube-like formation; 2) VEGF_165_-enhanced phosphorylation of VEGFR-2. In capillary tube-like formation assays, the ability of bone marrow endothelial cells to form tube-like structures in the presence of exogenous VEGF_165 _+/- PlnDI was quantified. Under serum free conditions, the addition of soluble VEGF_165 _(positive control) and PlnDI demonstrated dose dependent increases in lengths of tube-like structures formed (Figure 4A-B and 1F). Optimal concentrations for VEGF_165 _[20 ng/ml] and PlnDI [12.5 μg/ml] increased tube-like formation 35% and 24%, respectively.

Studies employing PlnDI, pre-treated with either chondroitinase ABC and/or a heparinase cocktail suggests the ability of PlnDI to enhance tube-like formation is HS chain dependent (Figure 4C). Moreover, PlnDI activity is further enhanced when its CS chains are removed. Interestingly, PlnDI/VEGF_165 _mixtures combine to enhance tube-like formation 16% relative to VEGF_165 _alone (Figure 4D). The synergy between PlnDI and VEGF_165 _is PlnDI-HS chain dependent (Figure 4D). PlnDI protein core/VEGF_165 _mixtures produce tube-like structures indifferent from those by VEGF_165 _alone. Unexpectedly, heparin/VEGF_165 _mixtures do not synergize in this system (Figure 4E).

**Figure 5:**
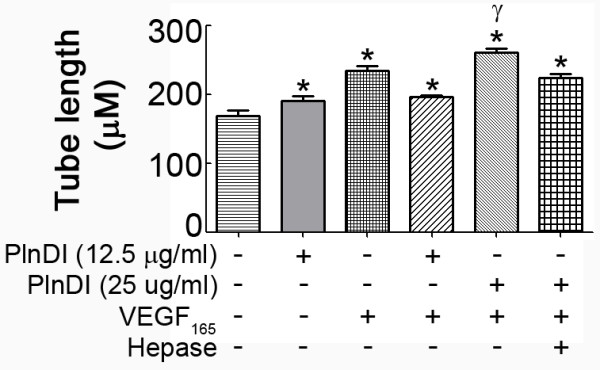
**In the absence of cell surface heparan sulfate increased PlnDI concentrations restore synergy with VEGF_165_**. Human bone marrow endothelial cells, in suspension, were treated with a heparinase cocktail, washed, then incubated with PlnDI [12.5 μg/ml] +/- VEGF_165 _[20 ng/ml] for 30 minutes prior to seeding in wells pre-coated with growth factor reduced Matrigel. After 18 h cells were fixed, stained with SYTO13, and tube length quantified. Both PlnDI and VEGF_165 _enhance capillary tube-like formation. A two fold increase in PlnDI concentration [25 μg/ml] is required for synergy with VEGF_165_. Data are presented as the mean lengths from nine areas of triplicate determinations ± SEM. (*) different from media; (γ) different from VEGF_165_. *p *< 0.05.

Since the presence of endogenous cell surface HS complicates the studies above, experiments employing bone marrow endothelial cells without cell surface HS were performed. Under these conditions, VEGF_165 _and PlnDI enhance tube-like formation (Figure 5); however, PlnDI/VEGF_165 _mixtures did not combine to further enhance the lengths of tube-like structures. Subsequent dose response studies suggested elevated concentrations of VEGF_165 _and PlnDI are required for maximal activity. Increasing PlnDI concentration two fold [25.0 μg/ml] restored synergy with VEGF_165 _in a PlnDI-HS chain dependent manner (Figure 5).

**Figure 6:**
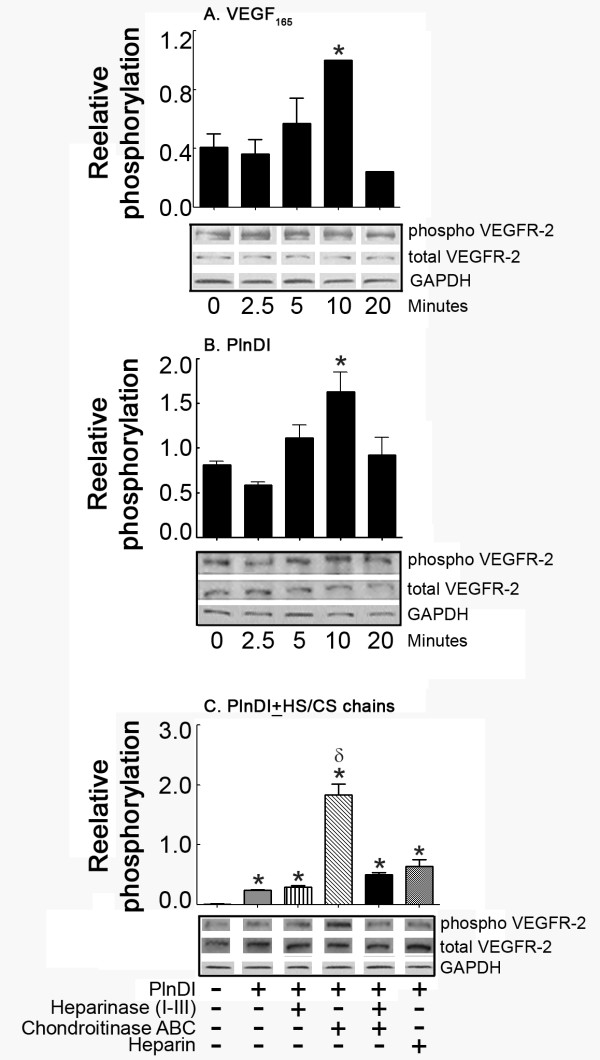
**PlnDI stimulates VEGFR-2 (Tyr-951) phosphorylation**. Human bone marrow endothelial cells without cell surface HS were incubated with VEGF_165 _or PlnDI for 0, 2, 5, 10 and 20 minutes. Cell lysates were analyzed for VEGFR-2 phosphorylation by Western blot using anti-phospho and total VEGFR-2 tyrosine residue 951 specific antibodies. Time dependant increase in VEGFR-2 phosphorylation induced by: (A) VEGF_165 _and (B) PlnDI. (C) PlnDI induced VEGFR-2 phosphorylation: Requirement for CS and HS chains. Exogenous heparin served as a positive control and GAPDH as loading control. Horizontal bars represent the densitometric scanning of bands outlined in rectangular boxes. Data are presented as the mean density values (DV) from triplicate determinations ± SEM. (*) different from 0 min or media. (δ) different from PlnDI. *p *< 0.05.

Because the role of HS in heparin-binding growth factor activity may involve interactions between HS, ligand, and cell surface receptors, the ability of PlnDI-HS to modulate VEGF_165_-induced VEGFR-2 tyrosine phosphorylation was investigated by Western blot using VEGFR-2 (Tyr-951) specific antibodies. VEGFR-2 phosphorylation at Tyr-951 results in recruitment of several adapter proteins whose subsequent downstream signaling supports endothelial cell survival and migration [19]. To perform these studies, we employed bone marrow endothelial cells whose cell surface HS were first removed by exposure to heparinases. Under these conditions, the exogenous addition of PlnDI and VEGF_165 _(positive control) enhanced VEGFR-2 phosphorylation at Tyr-951 (Figure 6A-B). The signal intensity of phosphorylation increased over time, peaked after ten minutes, then returned to control levels after 20 minutes (Figure 6A-B). The addition of PlnDI, adorned with only HS chains, enhances Tyr-951 phosphorylation ~3 fold relative to intact PlnDI (Figure 6C). Studies employing PlnDI preparations pre-treated with mixtures of chondroitinase ABC and heparinase enzymes did not completely attenuate phosphorylation (Figure 6C). Heparin addition (positive control) also enhanced VEGFR-2 phosphorylation (Figure 6C).

**Figure 7:**
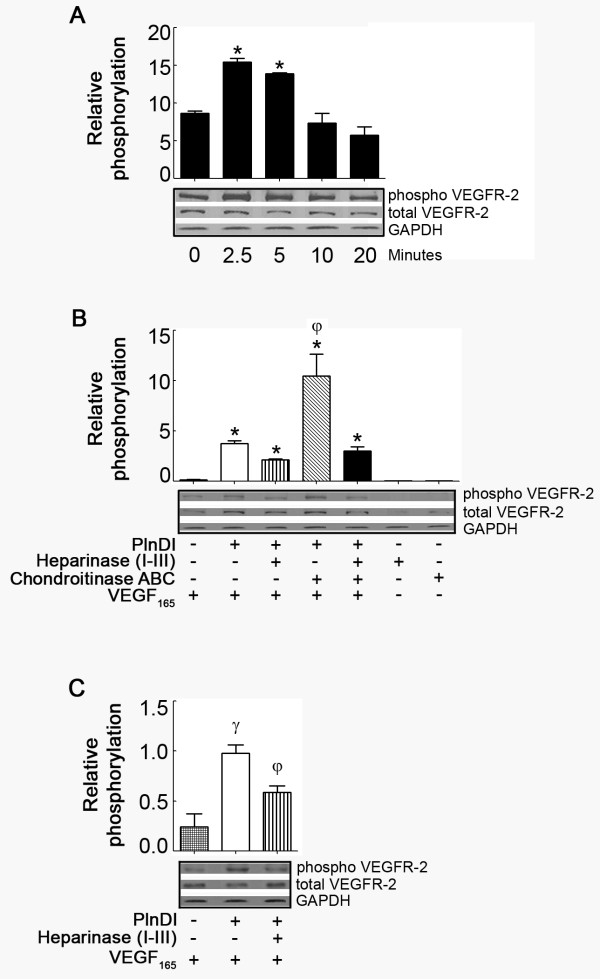
**PlnDI/VEGF_165 _mixtures enhance VEGFR-2 (Tyr-951) phosphorylation**. Human bone marrow endothelial cells without cell surface HS were incubated with PlnDI/VEGF_165 _mixtures for either 0, 2, 5, 10, or 20 min. Cell lysates were analyzed for VEGFR-2 (A and B) or Akt (C) phosphorylation by Western blot using anti-phospho and total VEGFR-2 (tyrosine residue 951) and Akt specific antibodies. (B) PlnDI/VEGF_165 _enhanced VEGFR-2 phosphorylation (at min 2.5): Requirement of CS and HS chains on PlnDI. Horizontal bars represent the densitometric scanning of bands outlined in rectangular boxes. GAPDH was assessed as a loading control. Data are presented as the mean of three independent experiments ± SEM. (*) different from 0 min; (γ) different from VEGF_165_; (φ) different from PlnDI+VEGF_165_. *p *< 0.05.

Relative to either alone, PlnDI/VEGF_165 _mixtures stimulate peak phosphorylation after only 2.5 minutes (Figure 7A vs. 6A-B). To identify the role of PlnDI-HS in modulating VEGF_165 _induced VEGFR-2 phosphorylation at Tyr-951, PlnDI preparations adorned with either CS, HS, or without GAGs were pre-mixed with VEGF_165_. The absence of HS chains on PlnDI reduced the signal intensity of phosphorylation 43% (Figure 7B). In contrast, preparations decorated only with HS chains enhance the signal intensity of phosphorylation ~3 fold (Figure 7B). The absence of CS and HS chains did not completely reduce the intensity of phosphorylation relative to control (VEGF_165_).

To determine if PlnDI/VEGF_165 _enhanced VEGFR-2 phosphorylation also promotes downstream signaling, blots were stripped then re-probed with antibodies specific for total and phosphorylated forms of Akt. PlnDI/VEGF_165 _mixtures enhance the signal intensity of phosphorylated Akt ~4 fold, relative to VEGF_165 _alone (Figure 7C), and ~40% of this activity is PlnDI-HS chain dependent.

**Figure 8:**
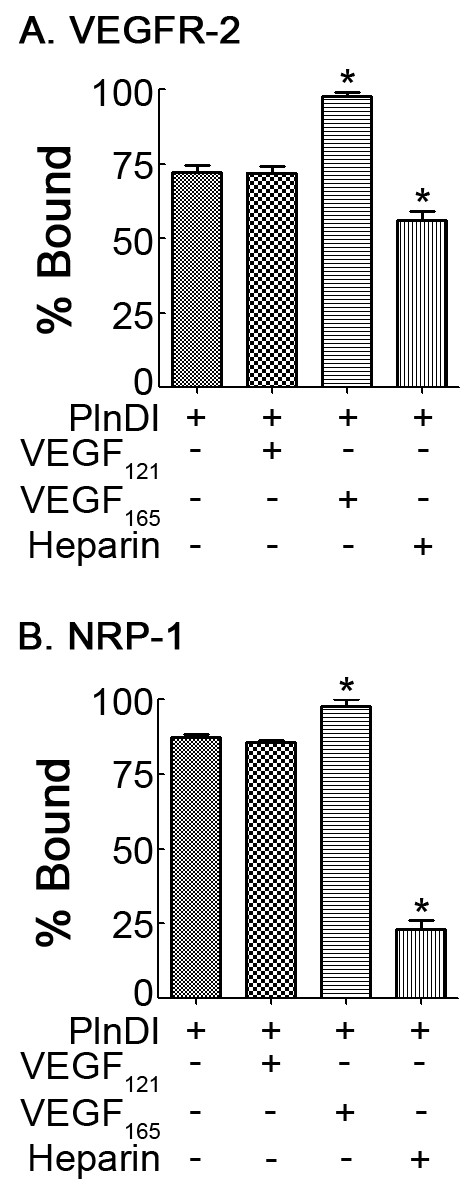
**PlnDI binds NRP-1 and VEGFR-2**. Recombinant NRP-1 and VEGFR-2 coated plates were incubated with PlnDI +/- VEGF_165 _or VEGF_121_. PlnDI binding was detected and quantified using an ELISA based approach that employed anti-PlnDI specific antibodies. For competitive inhibition assays, PlnDI was pre-incubated with heparin [1 μg/ml]. (A) PlnDI binding to VEGFR-2. (B) PlnDI binding to NRP-1. Data are presented as a percentage of total bound. (*) different from PlnDI. *p *< 0.05.

Since PlnDI may modulate phosphorylation via direct interactions with VEGFR-2 or a candidate co-receptor, we performed binding studies with immobilized recombinant VEGFR-2 and NRP-1. PlnDI binds VEGFR-2 and NRP-1 (Figure 8A-B); however, a higher percentage of PlnDI binds NRP-1. The presence of VEGF_165 _but not VEGF_121 _(negative control) enhances PlnDI binding to VEGFR-2 (27%) and NRP-1 (13%). The presence of heparin [1 μg/ml] reduces PlnDI binding to NRP-1 more than 60%. In contrast, PlnDI binding to VEGFR-2 was poorly competed away by heparin (Figure 8A-B).

## Discussion

**Figure 9:**
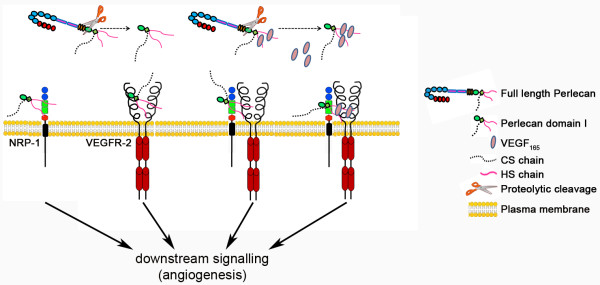
**Model: PlnDI interactions with the VEGFR-2 signaling complex**. N-terminal domain I of perlecan, unbound or bound to VEGF_165_, liberated by proteolytic cleavage (scissors) during extracellular matrix turnover stimulates angiogenesis by direct interactions with: 1) NRP-1; 2) VEGFR-2; and 3) NRP-1 and VEGFR-2. PlnDI may enhance VEGF_165 _stimulated pro-angiogenic events by stabilizing NRP-1/VEGF_165_/VEGFR-2 interactions.

For the first time, we have characterized the ability of recombinant PlnDI to bind VEGF_165 _and modulate its angiogenic activity, *in vitro*. We have shown that soluble forms of PlnDI are capable of modulating VEGFR-2 phosphorylation, as well as VEGF_165_-induced phosphorylation of VEGFR-2, and that the heparan sulfate glycosaminoglycan chains adorning PlnDI are important for these activities. Together, our observations suggest soluble forms of PlnDI may form and/or stabilize a complex between VEGF_165, _NRP-1, and VEGFR-2 to enhance angiogenic events and VEGFR-2 signaling in human bone marrow endothelial cells (summarized in Figure 9).

In contrast to our previous reports [17,20], the purity of PlnDI employed in the present investigation was enhanced by passage through a Sepharose CL-6B column. SDS-PAGE, Western blot and monosaccharide analysis suggest the molecular weight and GAG chain composition of PlnDI are similar to species previously characterized [20,21]. Moreover, these observations predict our preparation contains at least two species of PlnDI: one adorning predominately CS and the other predominately HS chains. Interestingly, the CS and HS disaccharide composition of PlnDI reported herein is different from species recently characterized by Whitelock et al. [22], as well as that reported for full length perlecan purified from bovine rib growth plate cartilage, HUAEC and RT101 cell lines [23-25]. These differences could be due to: 1) cell culture conditions; 2) approaches for purification; and 3) approaches employed for disaccharide analysis. Regardless, since fewer 4-sulfated CS residues and more 2-sulfated and 6-sulfated HS residues were identified it is reasonable to conclude that the function of PlnDI employed herein is distinct from forms previously reported. Indeed, subtle variations in HS substructure profoundly affect heparin-binding growth factor and receptor interactions, and thus the activity of perlecan [26-28].

While the role(s) of HS chains on perlecan have been most widely investigated with regard to regulation of FGF-2 activity [29,30], few studies have reported on perlecan-VEGF_165 _interactions [5,6,22]. Moreover, the GAG modifications required specifically for perlecan-VEGF_165 _interactions have not been described. Nevertheless, studies with heparin/HS suggest 2-*O*- and 6-*O*- sulfation is important for VEGF binding and activity [31-33]. Although the abundance of 2*-O*- and 6*-O-*sulfation on PlnDI-HS suggests it harbors the capacity to interact with VEGF_165_, a correlation between VEGF_165 _affinity and abundance of a particular disaccharide or the overall level of HS sulfation has not been observed [31]. Thus, growth factor binding is likely determined by HS domain organization (i.e., length of sulfation and transition domains, as well as their placement along the chain). Since HS chains on recombinant PlnDI are likely to be short (8-10 kDa) relative to those on tumor-derived perlecan (30-70kDa) [21,34,35], we predict 48 residues comprise a single HS chain on PlnDI (based on the molecular weight of repeating units of glucuronic acid and *N*-acetylglucosamine). Moreover, since six or seven oligosaccharide residues are sufficient to fully occupy the HS binding site for VEGF_165 _[31], we further predict that six VEGF_165 _binding sites (maximally) may be available on each HS chain attached to PlnDI.

The HS dependent binding of VEGF_165 _to immobilized PlnDI described herein is consistent with recent reports [5,6]. In contrast, a new communication has reported PlnDI does not bind immobilized VEGF_165 _[36]. We suspect the concentration and/or the disaccharide composition of PlnDI employed therein may account for the contrasting observations. Our studies with PlnDI in solution suggest VEGF_165 _binding to PlnDI in solution is not only HS but pH dependent. The marked reduction in VEGF_165 _binding to PlnDI under acidic conditions, a novel observation, is consistent with previous publications describing the attenuation of VEGF_165 _binding with low concentrations of heparin under acidic conditions, and its potentiation at neutral pH [14,37].

To identify the ability of soluble, exogenous PlnDI to modulate VEGF_165 _activity, *in vitro*, tube-like formation studies were performed with human bone marrow endothelial cells seeded on growth factor reduced (GFR) Matrigel. We hypothesized that PlnDI/VEGF_165 _mixtures would enhance the lengths of tube-like structures formed over VEGF_165 _alone. While our observations support this hypothesis, we were surprised that PlnDI addition, alone, also enhanced the length of tube-like structures. Given our experimental approach, the enhancement of tube-like formation by soluble, exogenous, PlnDI may also reflect interactions with other matrix molecules (i.e., fibronectin and laminin) and heparin-binding growth factors present in GFR Matrigel reported to interact with PlnDI [38]. This possibility, however, should not discount the ability of exogenous PlnDI to interact directly with human bone marrow endothelial cells, or the possibility that the presence of heparin-binding molecules and growth factors may even mask the full activity of PlnDI.

Interestingly, under conditions where bone marrow endothelial cells were pre-treated with a heparinase cocktail, the additive effect of PlnDI/VEGF_165 _mixtures on tube-like formation was not observed unless the concentration of PlnDI was increased two fold. While these observations suggest PlnDI-HS chains can modulate VEGF_165 _activity, *in vitro*, heparin/VEGF_165 _mixtures (positive control [14,32]), did yield similar results. We remain puzzled by this observation since heparin/VEGF_165 _mixtures combine to enhance VEGFR-2 phosphorylation, suggesting heparin is active in our system.

At the cellular/receptor level, we analyzed VEGFR-2 auto-phosphorylation to identify requirements for PlnDI modulation of VEGF_165 _activity, *in vitro*. While both VEGFR-1 and VEGFR-2 contribute to VEGF induced signals, VEGFR-2 dominates VEGF induced mitogenic and angiogenic responses in endothelial cells [11,12]. Of the six tyrosine phosphorylation sites identified on the intracellular domain of VEGFR-2, we report on one associated with endothelial cell survival and migration [39]. Together, our observations suggest exogenous soluble PlnDI, alone, can stimulate VEGFR-2 phosphorylation at Tyr-951. Moreover, PlnDI fragments harboring only HS chains further enhance VEGFR-2 phosphorylation, suggesting the presence of CS chains masks activity. These studies importantly extend those recently reported for full length perlecan [6] by demonstrating delivery of PlnDI or co-delivery with VEGF_165 _are sufficient to enhance VEGFR-2 phosphorylation, and promote downstream signaling (i.e., increased Akt phosphorylation). Given our approach (i.e., the use of cells in suspension), our observations suggest PlnDI/VEGF_165 _mixtures enhance survival signaling (increased Akt phosphorylation) of human bone marrow endothelial cells, *in vitro*. Consistent with this conclusion, our unpublished observations suggest VEGFR-2 phosphorylation at Tyr-1175 and Tyr 1214, and phosphorylation of p38 MAPK, Erk1/2 (events associated with endothelial cell proliferative and migratory states) [39], are unaltered.

Finally, to determine if PlnDI has the capacity to bind and modulate the activity of VEGFR-2 directly, we performed PlnDI binding studies against immobilized VEGFR-2, and NRP-1. Outcomes from these studies suggest PlnDI-HS chains, similar to heparin/HS, harbor the capacity to interact with VEGFRs and co-receptors [15,32,40], and enhance VEGFR-2 signaling [41]. We suspect PlnDI-HS chain binding to NRP-1 occurs via its heparin binding domain [15]. In contrast, PlnDI binding to VEGFR-2 is less dependent on HS chains. Heparin concentrations up to [100 μg/ml] did not appreciably alter binding (unpublished observations). Interestingly, the presence of VEGF_165 _enhances PlnDI binding to VEGFR-2, suggesting the formation of a complex between PlnDI/VEGF165/VEGFR-2 is possible. Our observations also suggest that modulation of VEGFR-2 signaling by PlnDI may involve complex interactions with more than one ligand.

## Conclusion

The findings presented herein demonstrate exogenous, soluble, recombinant PlnDI is sufficient to bind and modulate the activity of the VEGFR-2 signaling complex via HS interactions, *in vitro*. Moreover, PlnDI may have activities independent of those with heparin-binding growth factors in supporting tube-like formation, *in vitro*. Figure 9 provides a simplified visual depiction of how PlnDI may impact angiogenic events in the absence or presence of VEGF_165_. PlnDI unbound or bound to VEGF_165 _is liberated via cleavage within its SEA module [42] or the single immunoglobulin G-like region of domain II [43,44] during matrix turnover, wound healing, or disease progression. In the absence of VEGF_165, _PlnDI-HS may bind to NRP-1, VEGFR-2, or support complex formation with both to signal downstream angiogenic events. When VEGF_165 _is present PlnDI interactions with NRP-1 and VEGFR-2 are optimized, leading to enhanced downstream signaling and angiogenesis.

## Methods

### Materials

Recombinant human VEGF_165_, VEGFR-2, NRP-1, and anti-VEGF_165 _monoclonal antibodies were procured from R&D systems, Inc. (Minneapolis, MN). Growth factor reduced Matrigel was purchased from BD Biosciences (San Jose, CA). Goat polyclonal antibodies to GAPDH were purchased from Genscript (Piscataway, NJ). Rabbit polyclonal antibodies for phospho- and total- VEGFR-2, and Akt were purchased from Santa Cruz Biotechnology (Santa Cruz, CA) and Cell Signaling (Danvers, MA), respectively. Anti-Perlecan domain I monoclonal antibodies (CSI 001-71) were purchased from the Antibody Shop (Denmark). Anti-Perlecan domain IV antibodies were purchased from Millipore (Temecula, CA). Heparin, heparinase I, II and III and protease free chondroitinase ABC were purchased from Sigma (St. Louis, MO). Heparitinase II enzyme, 3G10 antibodies, and unsaturated heparan/heparin-disaccharide standards were purchased from Seikagaku Corp (Japan).

### Cell Culture

Human bone marrow endothelial cells, provided by Dr. G Almeida-Porada (University of Nevada, Reno, [45]), were cultured in M199 media supplemented with endothelial cell growth supplement (R&D systems Inc, Minneapolis, MN), 10% (v/v) heat-inactivated FBS, 1% (v/v) penicillin/streptomycin, 2 mM glutamax and heparin (15 U/ml). Cells were sub-cultured when 80-90% confluent using 0.05% (v/v) trypsin/EDTA. All cultures were maintained at 37°C in a humidified 5% CO_2 _atmosphere.

### Sepharose CL-6B enrichment of Recombinant PlnDI

Recombinant perlecan domain I (PlnDI) was prepared as described previously [17]. PlnDI was enriched by passage through a Sepharose CL-6B column (1 × 50 cm), pre-equilibrated with 50 mM Tris-HCl buffer, pH 8.6 containing 6 M guanidine-HCl and 0.5 M NaCl. Fractions were assayed for uronic acid by carbazole method [46], and protein by micro BCA assay (Pierce, Rockford, IL). PlnDI purity was assessed by SDS-PAGE (i.e., Alcian blue and Coomassie blue staining) and Western blotting (see below).

### Western Blotting

PlnDI (25 μg), untreated or pre-digested with heparinase cocktail (mixture of heparinases I, II and III, 2.5 Sigma units each) and/or chondroitinase ABC, were electrophoresed on 3-8% Tris-acetate gels (Invitrogen, CA), then transferred to nitrocellulose. Membranes were probed with anti-PlnDI monoclonal antibodies diluted (1:200) in phosphate buffered saline (PBS) with 0.1% (v/v) Tween-20 (PBST), containing 3% (w/v) BSA. Primary antibodies were detected with anti-mouse IgG secondary antibodies conjugated to peroxidase and visualized by incubation with enhanced chemiluminescence reagent (ECL, GE Healthcare), and exposure to film.

### Chondroitinase ABC and Heparinase digestion

For chondroitinase ABC digestion PlnDI (25 μg) was incubated with chondroitinase ABC (20 mU) in 25 μl of 100 mM/L Tris-HCl, pH 8.0, containing 30 mM/L sodium acetate and 0.01% (w/v) BSA at 37°C for 5 hours. For heparinase digestion, PlnDI was incubated with a heparinase cocktail in 25 μl of PBS containing 4 mM CaCl_2 _and protease inhibitors for 12 hours at room temperature.

### Immunoassays

Solid phase binding assays were performed as described previously [17]. For solution phase binding assays, PlnDI (5 μg) untreated, or pre-digested with a heparinase cocktail and/or chondroitinase ABC was pre-incubated with 20 ng of VEGF_165 _in PBS containing 3% (w/v) BSA, or 25 mM HEPES at either pH 8.0, 7.0, or 6.0 [37], or 50 mM Tris-HCl (pH 8.0), PBS (pH 7.0), 50 mM sodium acetate (pH 6.0) for 1 hr at room temperature. Samples were subsequently blotted onto nitrocellulose, and blocked. Bound VEGF_165 _was detected with anti-VEGF_165 _antibodies (1 μg/ml in 3% (w/v) BSA in PBST). Primary antibodies were detected with anti-mouse IgG secondary antibodies conjugated to HRP and visualized as described for Western blotting. Binding was quantified by densitometry and expressed as mean density values (DV) from triplicate assays. Specific binding was determined by subtracting VEGF_165 _background from total bound [18].

### Capillary Tube-like Assay

Growth factor reduced (GFR) Matrigel was added to wells of ice-cold 96-well plates (70 μl/well) for 6 seconds. Excess was removed, leaving a thin coating. Plates were incubated for 6 minutes on ice, 20 minutes at room temperature, and finally warmed for 20 minutes at 37°C. Bone marrow endothelial cells were seeded (6,500 cells/well) in serum free RPMI 1640 media containing 1% (w/v) penicillin/streptavidin, 2 mM glutamax without growth supplements. After cell attachment, the media was replaced with media containing one or more supplements [i.e., PlnDI (12.5 μg/ml), untreated or pre-digested with a heparinase cocktail and/or chondroitinase ABC, heparin (4.0 μg/ml), VEGF_165 _(20 ng/ml)]. For assays conducted in the absence of cell surface heparin sulfate, human bone marrow endothelial cells were cultured for 15 minutes under serum free conditions in RPMI 1640 media supplemented with heparinase cocktail [32]. Such treatments temporarily remove more than 95% of cell surface HS. Prior to seeding cells were washed twice with RPMI 1640 media.

To quantify tube-like formation cells were fixed (4% (v/v) paraformaldehyde) after 18 h, stained (SYTO13, Invitrogen, CA), then photographed with a SPOT CCD camera affixed to an inverted microscope equipped for epifluorescence. Nine random fields, representing 80% of each well, were analyzed for three angiogenic parameters: average tube length (defined as three or more cells connected lengthwise, and exceeding 100 μm in length; [47], number of tube-like structures, and the number of branch points, using Image J software (NIH). When several tube-like structures merged together or branched, the total length was calculated as the sum of the individual branches. All tube-like formation studies were conducted in quadruplicate wells, and repeated at least three times. Since the outcomes of each angiogenic parameter were similar only average tube length is reported. Note: All supplement concentrations employed herein are optimal, and were determined empirically over a broad range. As a control for enzyme activity, assays were also conducted with supplements containing heat inactivated chondroitinase ABC and/or heparinase cocktail.

### Receptor Phosphorylation studies

Bone marrow endothelial cells, cultured to 80% confluence, were rinsed twice with serum free media, and then cultured for 24 hrs under serum free conditions. Cells were pre-incubated with a heparinase cocktail to remove cell surface heparan sulfate (as described above) then incubated with serum free media containing supplements (PlnDI, VEGFs, and heparin, as described above) for either 0, 2.5, 5, 10 or 20 minutes. After washing (ice-cold PBS), the cells were lysed [10 mM Tris-HCl, buffer pH 7.4 containing 140 mM NaCl, 0.2% (v/v) Triton X-100, 1.5 mM EDTA, 1 mM Na_3_PO_4_, 25 mM NaF, and 1 mM Na_3_VO_4_, protease inhibitors (Roche Diagnostics, Lewes, UK)], and total protein concentrations determined (micro BCA assay). For Western blotting, 30 μg of each sample was loaded onto 7% Tris-acetate gels, electrophoresed under reducing conditions, then transferred onto nitrocellulose. Membranes were probed with anti-phospho-VEGFR-2 (tyrosine residue (Tyr)-951), phospho-Akt, anti total-VEGFR-2, total-Akt, and anti-GAPDH antibodies. Primary antibodies were detected and visualized as described for Western blotting.

### Enzyme Linked Immunosorbant Assay

Recombinant proteins (NRP-1 and VEGFR-2) were allowed to bind overnight (4°C) in 96-well plates (100 ng/well; Maxi Sorp, Nunc). After several washes and blocking with PBS containing 3% BSA, PlnDI (5 μg/well) with or without VEGF_121_, VEGF_165_, or heparin [0.01-1000 μg/mL] was added. After 2 h, and several washes with PBS containing 0.05% Tween 20, the plates were incubated with anti-PlnDI antibodies (1:500 dilution) for 1 h. Primary antibodies were detected with anti-rabbit IgG secondary antibodies conjugated to HRP (1:8000). Each well was subsequently incubated with tetramethylbenzidine (KPL; 100 μl/well) for 10 min under gentle agitation. Color development was stopped with 50 μL of 0.5 N H_2_SO_4_. Binding was quantified by measuring absorbance at 450 nm. Unless indicated, all incubations were performed at room temperature.

### Monosaccharide analysis

As done previously [46], PlnDI (20 μg) was hydrolyzed with 4 M HCl at 100°C for 6 h, then dried in a Speed-Vac. Residues were dissolved in HPLC grade water then analyzed on a CarboPac PA1 high pH anion-exchange column (4 × 250 mm) using Dionex BioLC HPLC coupled to a pulse amperometric detector.

### Disaccharide composition analysis of GAG chain

As done previously [48], CS disaccharides, released from PlnDI (20 μg) following digestion with chondroitinase ABC [(20 mU) in 50 μl of 100 mM Tris-HCl, 30 mM NaOAc, pH 8.0, containing 0.01% (w/v) BSA at 37°C for 5 h] were analyzed by HPLC on the 4.6 × 250 mm amine-bonded silica PA03 column. Unsaturated HS disaccharides released from PlnDI following digestion with heparinase cocktail were analyzed as above. Commercially prepared bovine tracheal CS disaccharide standards (ΔDi-0 S, ΔDi-4 S and ΔDi-6S), and unsaturated heparan/heparin-disaccharide standards were used to determine standard migration positions and for quantitation.

### Statistical analysis

All experiments were conducted in triplicate, repeated at least three times, and analyzed by two-tailed paired Student's *t-*test using GraphPad Prism version 5.0 for Windows (San Diego California USA). Differences were considered significant at *P *< 0.05. All results are presented as means ± standard error of the mean.

## Abbreviations

CS: chondroitin sulfate; GAG: glycosaminoglycan; HSPG: heparan sulfate proteoglycan; HS: heparan sulfate; NRP-1: Neuropilin 1; PlnDI: recombinant perlecan domain I; VEGF: vascular endothelial growth factor; VEGFR-2: vascular endothelial growth factor receptor-2; p-VEGFR-2: phosphor-VEGFR-2; t-VEGFR-2: total-VEGFR-2.

## Authors' contributions

AM performed all experiments described herein. RRG conceived the study, and participated in its design and coordination. All the authors contributed equally to data analysis, interpretation, and communication of the findings. All authors have read and approved the final version of the manuscript.
